# Insights into the genetics of menopausal vasomotor symptoms: genome-wide analyses of routinely-collected primary care health records

**DOI:** 10.1186/s12920-023-01658-w

**Published:** 2023-10-02

**Authors:** Katherine S. Ruth, Robin N. Beaumont, Jonathan M. Locke, Jessica Tyrrell, Carolyn J. Crandall, Gareth Hawkes, Timothy M. Frayling, Julia K. Prague, Kashyap A. Patel, Andrew R. Wood, Michael N. Weedon, Anna Murray

**Affiliations:** 1 Genetics of Complex Traits, University of Exeter Medical School, University of Exeter, Exeter, EX2 5DW UK; 2 Division of General Internal Medicine and Health Services Research, David Geffen School of Medicine at University of California, Los Angeles, CA 90024 USA; 3 Exeter Centre of Excellence for Diabetes Research, University of Exeter, Exeter, EX2 5DW UK; 4 Macleod Diabetes and Endocrinology Centre, Royal Devon and Exeter National Health Service Foundation Trust, Exeter, EX2 5DW UK

**Keywords:** Vasomotor symptoms, Menopause, Hormone replacement therapy, Genome-wide analyses, Genome-wide association study

## Abstract

**Background:**

Vasomotor symptoms (VMS) can often significantly impact women’s quality of life at menopause. In vivo studies have shown that increased neurokinin B (NKB) / neurokinin 3 receptor (NK3R) signalling contributes to VMS, with previous genetic studies implicating the *TACR3* gene locus that encodes NK3R. Large-scale genomic analyses offer the possibility of biological insights but few such studies have collected data on VMS, while proxy phenotypes such as hormone replacement therapy (HRT) use are likely to be affected by changes in clinical practice. We investigated the genetic basis of VMS by analysing routinely-collected health records.

**Methods:**

We performed a GWAS of VMS derived from linked primary-care records and cross-sectional self-reported HRT use in up to 153,152 women from UK Biobank, a population-based cohort. In a subset of this cohort (*n* = 39,356), we analysed exome-sequencing data to test the association with VMS of rare deleterious genetic variants. Finally, we used Mendelian randomisation analysis to investigate the reasons for HRT use over time.

**Results:**

Our GWAS of health-records derived VMS identified a genetic signal near *TACR3* associated with a lower risk of VMS (OR=0.76 (95% CI 0.72,0.80) per A allele, *P*=3.7x10^-27^), which was consistent with previous studies, validating this approach. Conditional analyses demonstrated independence of genetic signals for puberty timing and VMS at the *TACR3* locus, including a rare variant predicted to reduce functional NK3R levels that was associated with later menarche (*P* = 5 × 10^–9^) but showed no association with VMS (*P* = 0.6). Younger menopause age was causally-associated with greater HRT use before 2002 but not after.

**Conclusions:**

We provide support for *TACR3* in the genetic basis of VMS but unexpectedly find that rare genomic variants predicted to lower NK3R levels did not modify VMS, despite the proven efficacy of NK3R antagonists. Using genomics we demonstrate changes in genetic associations with HRT use over time, arising from a change in clinical practice since the early 2000s, which is likely to reflect a switch from preventing post-menopausal complications in women with earlier menopause to primarily treating VMS. Our study demonstrates that integrating routinely-collected primary care health records and genomic data offers great potential for exploring the genetic basis of symptoms.

**Supplementary Information:**

The online version contains supplementary material available at 10.1186/s12920-023-01658-w.

## Introduction

Vasomotor symptoms (VMS) in postmenopausal women include hot flashes and night sweats, which can have debilitating and long-lasting effects on quality of life, with 10% of women experiencing VMS for up to 12 years [1]. Hormone replacement therapy (HRT) is the most effective treatment for VMS around menopause but its popularity amongst the public and health professionals declined following the publication of adverse outcome data [2] and it is contraindicated in some women, such as those who have had breast cancer. Consequently, there is great interest in identifying non-oestrogen drug treatments for VMS.

VMS result from dysfunction of thermoregulation by the hypothalamus and autonomic thermoregulatory system caused by increased neurokinin B (NKB) /neurokinin 3 receptor (NK3R; also known as the neuromedin-K receptor or tachykinin receptor 3) signalling in response to decreased circulating oestradiol levels, as shown in vivo in animal models [3, 4]. Four Phase 2 clinical trials have demonstrated that administration of a NK3R receptor antagonist results in a clinically significant reduction in hot flushes comparable to that achieved by HRT [5–8]. Such NK3R receptor antagonists are currently in Phase 3 clinical trials and have not yet come to market [9]. Neurokinin signalling also plays an important role in controlling the onset of puberty [10], with genetic variants that knock out the *TACR3* gene (which encodes NK3R) resulting in pubertal failure in homozygous carriers [11, 12] and delayed puberty in heterozygous carriers [13]. A genome-wide association study (GWAS) in 17,695 women (12,276 with VMS) identified a genetic signal for VMS in the *TACR3* gene region [14], further replicated in a GWAS of oestrogen-replacement use [15]. Additionally, population-based genome-wide association studies (GWAS) have identified several more common genetic variants in or near the *TACR3* gene associated with normal variation in age at menarche [16].

We aimed to improve understanding of the genetic basis of VMS through genomic analyses. Few cohort studies with genetic data have collected self-reported information on VMS, thus we adopted a novel approach by deriving this phenotype from primary care health records in 92,028 women in UK Biobank [17, 18], a five-fold increase in sample size.

## Methods

### Phenotype definitions

Between 2006 − 2010, UK Biobank recruited over 500,000 individuals aged 37 − 73 years from across the UK who answered detailed questions about themselves, had measurements taken and provided blood, urine and saliva sample samples, and for whom linked health records data are available [18]. We identified women with VMS for inclusion in our primary analyses from linked primary care records, which are available for ~ 45% of the cohort and capture participants’ contact with health care professionals working at UK general practices (family physicians) over their lifetime to ~ 2017. Cases (*n* = 14,261) were women with one or more clinical events in the linked primary care records (e.g. symptoms, history or diagnosis) containing any of 33 relevant codes for menopausal VMS (Supplementary Methods; Supplementary Table 1). These codes were identified by reviewing 970 Read v2 and CTV3 codes with descriptions containing the full and partial words “flush”, “flash”, “meno”, “sweat”, “vaso” for relevance and frequency of use in females in UK Biobank. Controls (*n* = 77,767) were women who were included in the linked primary care records (identified through having a primary care registration record without a relevant VMS code), and aged 50 and over at the baseline UK Biobank assessment and so were likely to be experiencing the menopause transition and thus at risk of VMS.

For secondary analyses we derived proxy phenotypes for VMS based on self-reported HRT use (Supplementary Methods, Supplementary Table 2). To explore the effect of the publication of adverse health outcomes relating to HRT, we further subdivided the ever taking HRT group into those who took HRT before and after 2002 to reflect the year of publication of the initial findings of the Women’s Health Initiative HRT trial [19] and estimated the proportion of women taking HRT before and after 2002 (Supplementary Methods, Supplementary Table 2).

### Genome-wide association study analyses

Genotyping of the UK Biobank study was performed centrally [17]. Two genotyping arrays with over 95% common marker content were used to genotype the individuals; the Affymetrix Axiom UK Biobank array (~ 450,000 individuals) and the UKBiLEVE array (~ 50,000 individuals). This dataset underwent extensive central quality control and genotype imputation using 1000 Genomes Phase 3/UK10K and Haplotype Reference Consortium reference panels. We based our study on 451,099 individuals who we identified as being of European descent, as described previously [20].

We carried out genome-wide analyses using BOLT-LMM v2.3 [21], which uses a mixed linear model to account for relatedness and ethnicity. We tested 17 million genetic variants with minor allele frequency (MAF) > 0.1% and imputation quality > 0.3. Association testing was based on an additive model and was adjusted for genotyping chip and release of the data, recruitment centre and age (Supplementary Methods). Quantitative traits were inverse-rank normalised, to ensure that residuals were normally distributed. Genome-wide significant variants had *P* < 5 × 10^–8^. For case–control traits we transformed effect sizes (*β*) on the quantitative scale to odds ratios (using the transformation ln(OR) = *β*/(*μ* × (1-*μ*)), where *μ* is the fraction of cases) and confirmed associations using a Fisher’s exact test (Supplementary Methods). We performed case − control matched analyses of HRT use to account for different age distributions (Supplementary Methods). Independent signals were more than 500 kb from the next most significant variant for the same phenotype and were uncorrelated (r^2^ measure of linkage disequilibrium (LD) < 0.5) with signals for other VMS phenotypes. Additionally, to identify further independent signals at the *TACR3* locus, we carried out conditional analysis by re-running the VMS GWAS including the genotype at the lead variant as a covariate.

We meta-analysed the results of the VMS GWAS with summary statistics from the European cohorts (GARNET and WHIMS) of Crandall et al. [14] (5,195 cases and 2,990 controls). Inverse-variance weighted meta-analyses were carried out in METAL [22] with genomic-control correction applied and we included variants with MAF > 0.1% and imputation quality > 0.3.

### *In silico* analyses and annotation of GWAS signals

To identify variants with functional or regulatory consequences, we looked up variants in LD with our lead genetic variant in Variant Effect Predictor (build 38) [23], Ensembl (Human (GRCh38.p13 release 100) [24], HaploReg v4.1 [25] and goDMC (http://mqtldb.godmc.org.uk/). We investigated eQTLs in r^2^ > 0.8 with the top hits in PsychENCODE [26] and GTEx v8 (https://www.gtexportal.org/home). We calculated the distance of variants in r^2^ > 0.8 with the lead genetic variant to canonical and alternative splice sites for *TACR3* using Intropolis [27]. LD was calculated from best guess genotypes for 1000 Genomes Phase 3/HRC imputed variants in ~ 340,000 unrelated UK Biobank participants of white British ancestry using PLINK v1.9 [28]. Manhattan and quantile − quantile plots were produced in R using the package “qqman” [29]. LocusZoom *v1*.4 [30] was used to plot the association statistics at individual loci.

### Exome-wide analyses

We carried out gene burden association testing of rare variants using exome sequencing data in 184,431 individuals of European ancestry from UK Biobank [31] (Supplementary Methods). Variants in CCDS transcripts were annotated using Variant Effect Predictor [23] and we identified loss-of-function (LOF) variants (stop-gain, frameshift, or abolishing a canonical splice site (-2 or +2 bp from exon, excluding the ones in the last exon)) deemed to be high confidence by LOFTEE (https://github.com/konradjk/loftee). We conducted gene-burden analyses using SAIGE-GENE [32] for age at menarche and REGENIE [33] for VMS (different software was used due to updated analyses pipelines for computational reasons), which both account for relatedness and ethnicity and are suitable for analyses with unbalanced case:control ratios. We included recruitment centre and age at baseline as covariates and tested the association of variants with MAF < 0.001 in *TACR3* (transcript ENST00000304883) in aggregate as well as individually (Supplementary Table 3).

### Tests of associations of variants in the *TACR3* region

We investigated whether puberty timing genetic variants in/near the *TACR3* gene were also associated with VMS. These variants included a rare protein truncating variant which in homozygous state leads to hypogonadotropic hypogonadism and in heterozygous state to delayed menarche (rs144292455 C>T p.W275X, chr4:104577415, MAF = 0.06%) [13, 34] and five genetic variants associated with age at menarche in GWASs (rs55784701 (chr4:104247262), rs3733632 (chr4:104640935), rs62342064 (chr4:104665972), rs115260227 (chr4:104774698), rs17035311 (chr4:106066293), smallest MAF 1.3%) within ~ 1.5 Mb of *TACR3* [16]. Genotypes were extracted from imputed data except for rs144292455, which is a rare variant and thus poorly imputed; for this variant we used directly genotyped data, which have previously been shown to be reliable [35]. We tested the associations of these variants individually, in combination and when adjusted for genotype of our lead VMS genetic variant using regression analyses. We performed logistic (binary traits) and linear (quantitative traits) regression in Stata v14.0/v16.0 in 379,768 unrelated individuals of European descent [36]. We regressed outcomes on genotype including the covariates genotyping chip and release of genotype data, recruitment centre, age and the first five genetic principal components (generated as described previously [36]).

### Mendelian randomisation analyses

We used Mendelian randomisation (MR) analyses to test whether earlier age at menopause was the cause of women taking HRT, or just correlated with HRT use. We constructed a genetic instrument for the exposure age at menopause from 56 published genetic variants discovered in a meta-analyses of ~ 70,000 women that was independent of UK Biobank data [37]. Outcomes were based on our GWAS in UK Biobank. In each MR test we assessed a number of widely used methods including inverse-variance weighted MR (IVW MR) and those more robust to pleiotropy (weighted median MR, penalised weighted median MR and MR–Egger [38, 39]) to address the assumption that alleles that influence the exposure do not influence the outcome via any pathway other than through the exposure. We used IVW MR as our primary analysis method and interpreted directionally-consistent results across the various methods as strengthening our causal inference.

### Heterogeneity in effects of genetic variants before and after 2002

Following the publication of data on adverse effects of HRT in 2002, we hypothesized that the primary reasons why women took HRT changed before and after this date, and that these differences would be reflected in genetic associations. To test this, for genetic signals identified for HRT phenotypes, we compared the association of the lead variant with ever using HRT before and after 2002 by comparing the direction and calculating a heterogeneity chi-squared *P*-value using the package “metan” in Stata v14.0/v16.0. We carried out Mendelian randomisation (as described) to test the association of genetically-instrumented age at menopause with HRT use before and after 2002.

## Results

### Validation of VMS phenotype

**Fig. 1:**
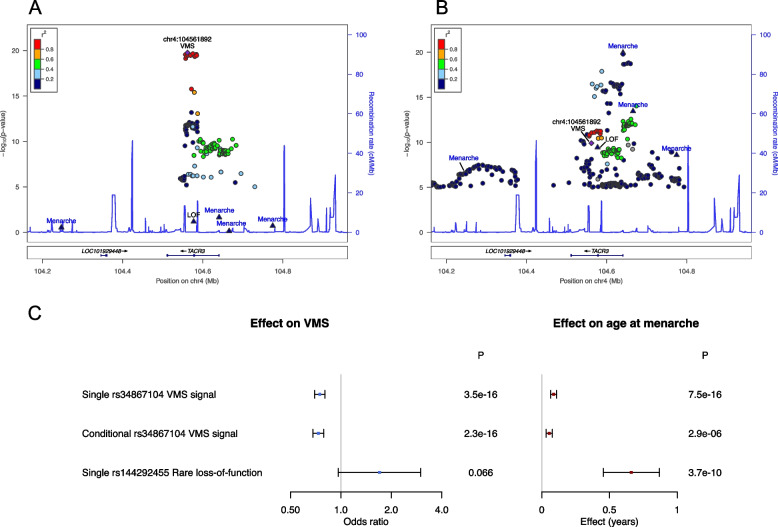
Genetic associations at the *TACR3* region: **A** Associations with VMS in UK Biobank. GWAS signals for age at menarche and the *TACR3* LOF allele (rs144292455 C > T) were not associated with VMS at *P* < 5 × 10^–8^ and show little correlation with the VMS signal (LD r^2^ < 0.2); **B** Associations with age at menarche in published GWAS. GWAS signals for age at menarche and the *TACR3* LOF allele (rs144292455 C > T) were strongly associated with age at menarche. The VMS lead variant shows association at *P* < 5 × 10^–8^ with age at menarche in this univariate analysis though was not identified as an independent signal in published age at menarche GWAS. **C** Univariate and conditional association analyses in UK Biobank. The VMS lead variant was associated with age at menarche at *P* < 5 × 10^–8^ in univariate analyses (“Single”) but this association attenuated in analysis adjusting for the genotypes of the age at menarche GWAS signals (“Conditional”). The *TACR3* LOF allele (rs144292455 C > T) was strongly associated with later age at menarche but not VMS. Notes: Variants shown are within ± 400 kb of rs34867104, the lead variant for VMS, and LD r^2^ shown is with rs34867104. Labelled variants are four variants associated with age at menarche within this region (triangles) [16], VMS (purple diamond) and also the LOF variant rs144292455 (triangle). For clarity, other variants with *P* > 1 × 10^–5^ are not shown. Association statistics for rs144292455 were calculated in directly genotyped data in UK Biobank whereas all other statistics are from GWAS of imputed data. Association statistics for age at menarche GWAS are from published ReproGen meta-analyses excluding 23andMe [16]. Results presented in Fig. 1C are from analysis of directly genotyped data performed in 379,768 unrelated individuals adjusting for genetic principal components (Supplementary Table 5)

We identified 14,261 women with VMS and 77,767 controls from linked primary care records in UK Biobank for inclusion in our genome-wide association study (GWAS) of VMS (Table 1). From our GWAS we identified a single independent genetic signal (lead variant rs34867104) associated with lower odds of having VMS ((OR = 0.78 (95% CI 0.74,0.82) per AT allele; allele frequency (AF) = 5.5%; *P* = 1.7 × 10^–20^; imputation quality = 0.99) (Supplementary Fig. 1, Fig. 1A). Meta-analysis of the UK Biobank VMS GWAS with that from Crandall et al. [14] replicated the same single genetic signal in the *TACR3* gene (OR = 0.76 per A allele (95% CI 0.72, 0.80; *P* = 3.7 × 10^–27^) (Table 2) with little evidence of heterogeneity (*P* = 0.025), validating our primary care health records derived phenotype. No further independent signals at *P* < 5 × 10^–8^ were identified when we performed GWAS conditioning on the genotype at the lead variant (rs34867104). Lead variant rs34867104 is intronic in *TACR3* and *in silico* analyses found little evidence to suggest a biological mechanism for the GWAS signal. There were no non-synonymous variants (with effects on protein sequence) strongly correlated with the signal (LD r^2^ > 0.8), and there was little evidence for an effect on gene expression, regulation or splicing (Supplementary Results). However, based on clinical studies that have demonstrated that NK3R antagonists reduce VMS [5, 40, 41], *TACR3* is the most likely causal gene in this region.

**Table 1:** Descriptive statistics for demographic and reproductive characteristics of women included as cases and controls in GWAS of VMS phenotypes

	Vasomotor symptoms					Ever taken HRT				
	Cases		Controls		*P*	Cases		Controls		*P*
	N	Mean (SD) /	N	Mean (SD) /		N	Mean (SD) /	N	Mean (SD) /	
		% (n)		% (n)			% (n)		% (n)	
Age	14,261	56.08 (7.37)	77,767	60.55 (5.39)	< 1E-50	72,622	61.82 (5.09)	80,686	59.6 (5.81)	< 1E-50
Body mass index	14,209	27.25 (5.03)	77,489	27.31 (5.11)	0.20	72,371	27.04 (4.81)	80,417	27.00 (5.16)	0.15
Age at menarche	13,832	12.95 (1.6)	75,335	12.93 (1.57)	0.36	70,614	12.94 (1.57)	78,102	12.94 (1.54)	0.63
Age at natural menopause	4,956	50.17 (4)	42,920	50.47 (3.91)	3.0E-07	33,250	49.53 (4.33)	72,629	50.7 (3.74)	< 1E-50
Number of births	14,253	1.80 (1.15)	77,719	1.90 (1.16)	4.0E-21	72,583	1.91 (1.14)	80,640	1.85 (1.18)	7.0E-23
Number of pregnancies	13,977	2.31 (1.56)	76,437	2.34 (1.54)	0.018	71,437	2.39 (1.54)	79,382	2.29 (1.55)	3.9E-32
Townsend deprivation index	14,241	-1.51 (2.9)	77,673	-1.57 (2.89)	0.022	72,544	-1.57 (2.91)	80,607	-1.62 (2.87)	6.6E-04
Smoking status	14,087		76,901		5.4E-27	71,709		79,915		
- Never		56.2% (7,912)		58.6% (45,057)			53.1% (38,102)		61.8% (49,351)	
- Previous		33.8% (4,768)		34.1% (26,208)			38.8% (27,845)		31.3% (25,023)	
- Current		10.0% (1,407)		7.3% (5,636)			8.0% (5,762)		6.9% (5,541)	
Vasomotor symptoms	14,261		77,767			33,015	16.8% (5,533)	36,106	7.5% (2,717)	< 1E-50^*^
Ever taken HRT	8,250	67.1% (5,533)	60,871	45.1% (27,482)	< 1E-50^*^	72,622		80,686		
Used HRT before 2002	6,779	59.9% (4,062)	55,748	40.1% (22,359)	< 1E-50	57,740		80,686		
Used HRT after 2002	3,625	25.0% (908)	35,304	5.4% (1,915)	< 1E-50	6,640		80,686		

All variables are at baseline except for age at menopause, which was calculated from the the most recent available visit. Ever taken HRT was defined in postmenopausal women and was identified by the answer to: “Have you ever used hormone replacement therapy (HRT)?”. For categorical variables, N is the total number of cases or controls with information; Age at menarche excludes values < 9 and > 17 years; Age at natural menopause excludes values < 40 and > 60 years. Townsend deprivation scores range from negative values to positive values with smaller scores indicating less material deprivation. *P*-values are from a t-test for continuous variables and a chi-square test (contingency table) for categorical variables

^*^Indicates *P* value for chi-squared test for ever taken HRT vs vasomotor symptoms

**Table 2:** Genetic signals for VMS from primary GWAS analysis and secondary analyses of HRT proxy phenotypes

**Analysis**	**Variant id**	**Chr:pos (b37)**	**EA/OA (EAF)**	**Phenotype**^**a**^	**Effect (95% CI) per allele**	***P***	**Distance to nearest gene**
Primary discovery	rs34867104	chr4:104561892	AT/A (0.055)	VMS	OR = 0.78 (0.74,0.82)	1.7 × 10^–20^	*TACR3* (0)
Meta-analyses^b^	rs112390256	chr4:104575473	A/G (0.055)	VMS	OR = 0.76 (0.72,0.80)	3.7 × 10^–27^	*TACR3* (0)
Secondary discovery	rs34867104	chr4:104561892	AT/A (0.055)	Ever taken HRT	OR = 0.85 (0.82,0.87)	1.1 × 10^–26^	*TACR3* (0)
Secondary discovery	rs146705358	chr4:104579107	AGGAATGTGCACAT/A (0.036)	Age ended HRT	-0.07 (-0.1,-0.05)	2.8 × 10^–8^	*TACR3* (0)
Secondary discovery	rs200480420	chr6:135016903	A/AT (0.999)	Time taken HRT	-0.51 (-0.69,-0.33)	4.3 × 10^–8^	*LINC01010* (+ 191.7 kb)
Secondary discovery	rs7830431	chr8:10700317	G/A (0.597)	Age started HRT	0.03 (0.02,0.04)	2.9 × 10^–8^	*PINX1* (+2.9 kb)

*Chr* chromosome, *CI* confidence interval, *EA* effect allele, *EAF* effect allele frequency, *HRT* hormone replacement therapy, *OA* other allele, *OR* odds ratio, *pos* position, *VMS* vasomotor symptoms

^a^Units for age started, ended and time taken HRT are standard deviations of the inverse rank transformed phenotype

^b^Variant rs112390256 represents the same genetic signal in *TACR3* as that identified from the primary discovery analysis since it is in linkage disequilibrium with rs34867104 (LD r^2^ = 0.99), which was not analysed in the earlier study

### No effect on VMS of genetic variants predicted to reduce levels of NK3R

Using exome sequencing data available for 6,280 women with VMS and 33,076 controls we investigated genetic variants in *TACR3* with alleles predicted to result in no protein product in homozygous individuals (i.e. LOF variants). We tested the association of LOF allele rs144292455 C > T p.W275X (MAF = 0.06%) previously reported as associated with delayed menarche [13, 34] and all rare (MAF < 0.1%) LOF variants in *TACR3* in aggregate. There was little evidence of an association between *TACR3* LOF and VMS (rs144292455 *P* = 0.6, 8/41 carriers were VMS cases vs 6,272/39,315 non-carriers; aggregate LOF burden *P* = 0.9, 8/49 carriers were VMS cases vs 6,272/39,307 non-carriers), though *TACR3* LOF was associated with delayed age at menarche (rs144292455 *P* = 5.0 × 10^–9^, 105 carriers vs 98,386 non-carriers; aggregate LOF burden *P* = 8.6 × 10^–11^, 128 carriers vs 98,363 non-carriers) (Supplementary Table 4). All carriers of the LOF alleles were heterozygous and so would be expected to have reduced levels of TACR3 protein. Associations of rs144292455 with VMS and age at menarche were consistent when we repeated the analysis in a larger sample of directly genotyped chip data (18/79 carriers were VMS cases vs 11,832/76,335 non-carriers) (Supplementary Table 5). We further confirmed the independence of the VMS GWAS signal (rs34867104) and rs144292455 through conditional analyses (Supplementary Table 5).

### Independent genetics of puberty timing and VMS at *TACR3*

We investigated the association with VMS of five common/low frequency genetic variants (smallest MAF 1.3%) in/near *TACR3* which have been associated with puberty timing in GWAS [16]. These five variants were not associated with VMS (*P* > 0.02 for all) and showed little correlation with the VMS signal rs34867104 (LD r^2^ ≤ 0.12 for all) (Fig. 1A and B). The VMS signal (rs34867104) was associated with age at menarche (Fig. 1B, Supplementary Table 6) [16], but conditioning on the five genetic variants for age at menarche attenuated the association (*P* = 7.5 × 10^–16^ in single variant analyses; *P* = 2.9 × 10^–6^ in conditional analyses) (Fig. 1C, Supplementary Table 6), suggesting that rs34867104 does not represent an independent signal for menarche timing. Taken together with the analyses of LOF variants, our results suggest differences in the genetic architecture of age at menarche and VMS at the *TACR3* locus.

### HRT proxy phenotype reflects menopause timing

Next we hypothesised that we could increase the number of cases included in our analyses five-fold by employing menopausal HRT use as a proxy phenotype for VMS, since this phenotype is available in self-reported data and HRT is predominantly used to treat menopausal symptoms. We identified variant rs34867104 as the most strongly associated signal (Table 2) (for ever taken HRT, OR = 0.85 (95% CI 0.82,0.87) per AT allele, *P* = 1.1 × 10^–26^), with consistent results in age-matched sensitivity analyses (OR = 0.83 (95% CI 0.80,0.86)). Our proxy phenotype analyses resulted in a further 14 independent genetic signals associated with HRT (age started and time taking) that did not reach genome-wide significance in the VMS GWAS (all *P* > 0.05) (Supplementary Results, Supplementary Table 7). All of these 14 signals had imputation quality > 0.9. We explored previously reported associations for menopause timing at these signals to test the validity of this approach finding that 11 of the 14 signals represented associations with HRT as a consequence of early menopause (Supplementary Table 7). In contrast, the VMS signal at *TACR3* (rs34867104) was not associated with menopause timing. Three additional genetic signals near *C3orf43*, *LINC01010* and *PINX1* were not associated with menopause timing and represent putative VMS signals. To test whether there was a causal association between menopause timing and using HRT, we performed Mendelian randomisation analyses. A one-year genetically-instrumented earlier age at menopause was strongly associated with higher odds of taking HRT (OR = 1.10 (1.08,1.12), *P* = 1 × 10^–18^) (Supplementary Table 8) but showed little association with VMS (*P* > 0.05). Overall, these genetic analyses show that the HRT use phenotype does capture VMS, but not specifically.

### Genetics capture changes in characteristics of women using HRT

**Fig. 2:**
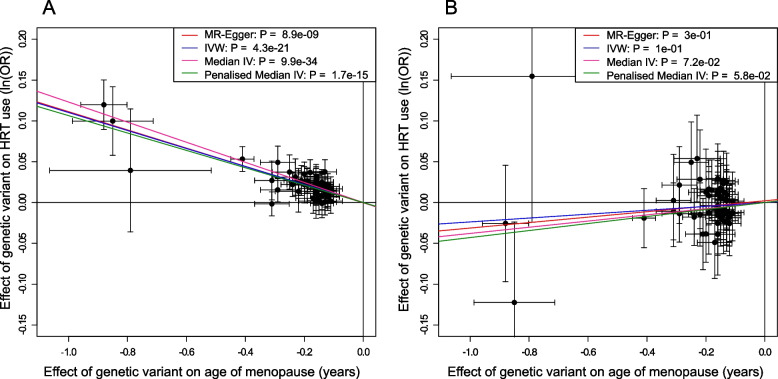
Effect of age at natural menopause on HRT use: **A** before 2002; and **B** after 2002. Mendelian randomisation analyses showing effect of genetic instrument for age at menopause on odds of using HRT

In UK Biobank we identified a change in the proportion of women using HRT from 49% before 2002 to 14% after 2002. We hypothesized that the publication of adverse trial results for HRT in 2002 would result in differences in the characteristics of women taking HRT before and after this time that would be reflected in genetic associations, while associations due to underlying biology of VMS should remain similar. The signals that were associated with HRT as a result of menopause timing had opposite effects on HRT use before and after 2002 (Supplementary Table 9), whereas the genetic signal in *TACR3* (rs34867104) was associated consistently over time (before 2002, OR = 0.85 per AT allele (95% CI 0.83,0.88), *P* = 1.6 × 10^–21^; after 2002, OR = 0.83 per AT allele (95% CI 0.77,0.89), *P* = 1.4 × 10^–6^; P test of heterogeneity = 0.48). Two of the putative VMS signals (rs201598433 near *C3orf43* and rs200480420 near *LINC01010*) also showed little evidence of heterogeneity in effects pre/post 2002 (Supplementary Table 9), strengthening the support for being candidate VMS signals (Table 2). By testing the association of genetically-instrumented age at menopause with HRT use before and after 2002, we found that a one-year genetically-predicted earlier menopause raised the odds of HRT use before 2002 (OR = 1.12, 95% CI = 1.10,1.13), but not after (OR = 0.98, 95% CI = 0.95,1.00) (Fig. 2), with concordant results in age-matched sensitivity analyses (Supplementary Table 8).

## Discussion

In this large study of menopausal VMS derived from primary care health records, we demonstrate the potential of routine health care data for deriving phenotypes not available in self-reported study data. We provide further evidence to support the role of *TACR3* in the genetic basis of VMS [14] and identified considerable phenotypic and genetic heterogeneity at the *TACR3* locus that should be explored further to provide important insight into potential therapies targeted at *TACR3*. We demonstrate that HRT phenotypes can be used as a proxy for VMS, provided that analyses consider the impact on genetic associations of major changes in the use of that therapy in routine clinical practice. Based on our results we suggest that HRT use post-2002 is more appropriate for capturing severe menopausal symptoms than pre-2002 use.

We replicated a genetic signal in *TACR3* associated with VMS [14], adding to genetic evidence for a role of NK3R (coded for by *TACR3*) in VMS. In postmenopausal women, oestrogen withdrawal results in hypertrophy of kisspeptin-neurokinin B-dynorphin secreting (KNDy) neurons in the infundibular nucleus of the hypothalamus, which increase mRNA expression of NKB, dynorphin, kisspeptin, substance P and ERα [42]. Studies in rodents have shown that increased levels of NKB result in more signalling through NK3R, stimulating pre-optic thermoregulatory areas of the hypothalamus but do not entirely exclude contributions of other KNDy peptides to thermoregulation (i.e. dynorphin, kisspeptin and substance P) [3, 4]. In humans, previous studies have demonstrated that infusion of NKB in pre-menopausal women induces menopausal VMS [41] and, in clinical trials, that NK3R antagonists reduce VMS in menopausal women [5, 40, 41], providing support for *TACR3* as the most likely causal gene in the GWAS locus. However, our *in silico* analyses found no additional evidence to confirm *TACR3* as the causal gene and we identified no reduction in VMS in 41 heterozygous carriers of a rare LOF genetic variant in *TACR3* (rs144292455) [34, 43] in whom NK3R expression should be reduced. Thus we cannot rule out the possibility that *TACR3* is not the causal gene for the VMS GWAS signal and that the GWAS signal is tagging a variant with an effect on another gene. Other published GWAS studies have identified significant signals in *TACR3* associated with testosterone levels (in males, and males and females combined) [44], and near *TAC3* (which codes for NKB) associated with age at menopause [45]. Further expanded genomic analyses of VMS and related phenotypes and the increasing availability of additional genomic data will allow these inconsistencies to be explored.

We provide evidence that the mechanism through which the genetic variants in *TACR3* influence VMS are distinct from effects of variants in this genomic region on puberty timing, consistent with known biology supporting distinct pathways. At puberty, NKB signalling is involved in the activation of Kiss1 neurons, resulting in pulsatile secretion of kisspeptin and consequently gonadotropin releasing hormone and luteinising hormone [10]. The rare variant in *TACR3* tested in our analyses (rs144292455) causes a premature stop codon (p.W275X) in the fifth transmembrane segment of the 465 amino acid NK3R and is predicted to be pathogenic and cause loss of function [34, 43]. Variant rs144292455 causes idiopathic hypogonadotropic hypogonadism in male homozygotes [34] and the rare allele shows additive effects, delaying menarche by 1.25 years in female heterozygotes [13, 35], an effect confirmed in our study cohort despite the lack of association with VMS. One explanation for these apparently contradictory results could be that complete inhibition of NK3R signalling [46] might be required to reduce VMS, and that the heterozygous women in our analyses still had sufficient NKB/NK3R signalling to cause VMS, resulting in no discernible reduction in symptoms. In contrast, we suggest that puberty timing appears to be sensitive to the amount of NKB/NK3R signalling. We were unable to test the effects of homozygous LOF of rs144292455 on VMS, as there were no such women in our study cohort. Common genetic variation associated with age at menarche at the *TACR3* locus is independent of rare variant rs144292455 [13] and was also not associated with VMS. We propose that changes in gene regulation and expression mediate the VMS phenotype, potentially through alterations in transcription factor binding, rather than the direct effects on NK3R, and that this should be explored in future studies.

We demonstrate an interaction between a major change in clinical practice and genetic determinants for HRT use, adding to the relatively small number of robust gene-by-environment interactions in the literature. Overall, women with earlier menopause were more likely to use HRT, but prior to 2002, earlier age at menopause increased the odds of HRT use but this relationship did not exist after 2002. In contrast, the genetic signal for VMS use in *TACR3* (rs34867104) remained associated with using HRT across the time period and was not associated with menopause timing. We suggest that prior to 2002, as recommended at the time, women took HRT as a routine treatment to relieve menopausal symptoms and also to prevent complications secondary to oestrogen depletion, for example, reduced bone mineral density. After 2002, we postulate that only women with severe VMS took HRT due to perceived risks that led to an aversion of women, health professionals and clinical guidelines to HRT [47]. It is surprising that earlier age of menopause was not associated with HRT use after 2002, as typically HRT will still be recommended until the average natural age of menopause for women with an early age of menopause. The lack of association of age at menopause with HRT use post 2002 highlights a possible potential under usage of HRT in the UK that requires further investigation. Current UK National Institute for Health and Care Excellence guidance is that HRT should be considered as a treatment for VMS [48] and our analysis suggests that HRT may now be underutilised among such women. Our findings suggest that genetic associations with HRT use that do not change over time are likely to be within the causal pathway for VMS.

Our analyses were carried out in UK Biobank, in which there are known biases towards healthy and more affluent individuals [49], in individuals who were similar to European genetic reference populations, highlighting a need for replication in other cohorts and ancestries. The impact of changes to clinical practice regarding HRT use may not be the same in all populations and future analyses must be mindful of other such gene by environment interactions. Currently, linked primary care data in UK Biobank are limited to approximately half the cohort and the coding of VMS depends on women consulting a GP with symptoms, and on these being recorded. Inclusion of women with VMS without a GP record as controls in our analyses will have resulted in reduced statistical power, but not false positives. The proxy phenotype of taking HRT did not distinguish between types of medication and is based on retrospectively collected questionnaire data, which may be subject to recall bias. However, another published study that analysed self-reported medication data in UK Biobank identified the same genetic signal for HRT use [15].

By identifying genetic associations with VMS and with the proxy phenotype HRT use we replicated a genetic signal for VMS in *TACR3* and identified a further two signals that require replication. We identified time-dependent genetic associations for HRT use that were associated with menopause timing but not VMS, providing insights into the factors driving treatments and allowing refinement of a proxy phenotype for VMS, i.e. HRT use after 2002. Despite the strong evidence for a role of *TACR3* in VMS from clinical trials of NK3R antagonist treatments, our study highlights limitations in current understanding that should be addressed in future studies to further benefit the development of therapies for these common, potentially life-changing symptoms.

### Supplementary Information

**Additional file 1: Supplementary Methods. Supplementary Results.** **Supplementary Figure 1.** Quantile-quantile and Manhattan plots for the GWAS of VMS in UK Biobank. **Supplementary Figure 2.** Quantile-quantile and Manhattan plots of the GWASs of HRT use.

**Additional file 2: Supplementary Table 1.** Read v2 and CTV3 codes used to identify women with vasomotor symptoms. **Supplementary Table 2.** Numbers of women included in the genome-wide analyses. **Supplementary Table 3.** Loss-of-function variants in *TACR3* identified in analysis of UK Biobank exome sequencing data. **Supplementary Table 4.** Results of gene burden and single variant analyses of *TACR3* in exome sequencing data from UK Biobank. **Supplementary Table 5.** Comparison of the effects on VMS and age at menarche of rs34867104 (GWAS signal) and rs144292455 (rare loss of function variant) in TACR3. **Supplementary Table 6.** Conditional analyses of variants in *TACR3* associated with VMS and age at menarche. **Supplementary Table 7.** Genetic signals identified by GWAS of HRT phenotypes. **Supplementary Table 8.** Results of Mendelian randomisation analyses of association of age at menopause with HRT use. **Supplementary Table 9.** Heterogeneity in effect of genetic variants on HRT use before and after 2002. **Supplementary Table 10.** PsychENCODE brain eQTL data for *TACR3*.

## Data Availability

All data used in the discovery analyses are available from UK Biobank on application at https://www.ukbiobank.ac.uk/enable-your-research/apply-for-access. Data from UK Biobank that are released to approved projects are de-identified with project specific identifiers assigned to individuals. The genome-wide summary statistics generated by the study are available on the GWAS Catalog (https://www.ebi.ac.uk/gwas) under accession ID GCST90267381.
